# Boron Neutron Capture Therapy (BNCT) for Cutaneous Malignant Melanoma Using ^10^B-p-Boronophenylalanine (BPA) with Special Reference to the Radiobiological Basis and Clinical Results

**DOI:** 10.3390/cells10112881

**Published:** 2021-10-26

**Authors:** Hiroshi Fukuda

**Affiliations:** Department of Radiology, Tohoku Medical and Pharmaceutical University, Sendai 983-8536, Japan; hirofuku@tohoku-mpu.ac.jp; Tel.: +81-22-290-8781

**Keywords:** radiobiology, BNCT, BPA, melanoma, clinical outcome

## Abstract

BNCT is a radiotherapeutic method for cancer treatment that uses tumor-targeting ^10^B-compounds. BNCT for cutaneous melanoma using BPA, a phenylalanine derivative, was first initiated by Mishima et al. in 1987. This article reviews the radiobiological basis of melanoma control and damage to normal tissues as well as the results of clinical studies. Experimental studies showed that the compound biological effectiveness (CBE) values of the ^10^B (n, α)^7^Li reaction for melanoma control ranged from 2.5 to 3.3. The CBE values of the ^10^B (n, α)^7^Li reaction for skin damage ranged from 2.4 to 3.7 with moist desquamation as the endpoint. The required single radiation dose for controlling human melanoma was estimated to be 25 Gy-Eq or more by analyzing the 50% tumor control dose data of conventional fractionated radiotherapy. From the literature, the maximum permissible dose to human skin by single irradiation was estimated to be 18 Gy-Eq. With respect to the pharmacokinetics of BPA in patients with melanoma treated with 85–350 mg/kg BPA, the melanoma-to-blood ratio ranged from 2.1–3.8 and the skin-to-blood ratio was 1.31 ± 0.22. Good local tumor control and long-term survival of the patients were achieved in two clinical trials of BNCT conducted in Japan.

## 1. Introduction

Boron neutron capture therapy (BNCT) is a radiotherapeutic method for cancer that uses boron-10 (^10^B), which efficiently absorbs thermal neutrons (cross section: 3830 barn) and releases α particles with high LET (230 keV/μm) through the ^10^B (n, α)^7^Li reaction. Because the range of the particle is only about 10 μm, which is close to the diameter of a cell, the thermal neutron irradiation causes significant damage only to cells that have taken up the tumor-seeking ^10^B-compound. Clinical trials of BNCT were first performed for the glioblastoma multiforme [1,2]. Subsequently, BNCT was also applied to malignant melanoma [3,4], and head and neck cancers [5,6,7]. In 2012, a clinical trial of BNCT using accelerator-based epithermal neutrons was initiated at the Kyoto University Reactor Institute, followed by clinical trial at the Southern Tohoku Hospital, Japan. On the basis of the results of these clinical trials, the Japanese government approved the health insurance coverage of BNCT for head and neck cancer in 2020.

Malignant melanoma is radioresistant and is one of the most treatment-resistant tumors. Mishima [8] was the first to propose BNCT for melanoma utilizing the specific melanin synthesis activity of melanoma cells. For this purpose, ^10^B-p-boronophenylalanine (BPA), which has a chemical structure similar to tyrosine, the precursor amino acid of melanin polymers, was reevaluated by Yoshino and Mishima and found to be a good melanoma-seeking ^10^B-compound [9]. BPA can be taken up by any kind of cancer cells as a result of enhanced cellular amino acid transport. It remains in melanoma cells more than in other cancer cells as it interacts with the melanin polymerization process of melanoma cells [10]. Interdisciplinary studies in many research fields are necessary for the successful clinical application of BNCT. Research in the field of radiation biology and radiation oncology is particularly important. In 1987, on the basis of the results of the experimental, technical, and preclinical studies on BNCT of melanoma [11,12,13,14,15,16,17], Mishima et al. applied BNCT with BPA for the first time in a patient with metastatic melanoma and obtained good local control of the tumor. Since then, until 2002, a total of 22 patients with melanoma were treated with BNCT [3,4,18,19,20,21,22,23,24], which provided good tumor control and longer patient survival. Hiratsuka also performed BNCT for eight cutaneous melanomas and obtained good results [25].

Although many reviews on BNCT for glioblastome using BSH have been published, there have been no comprehensive review on the BNCT of malignant melanoma using BPA. This article reviews the radiobiological characteristics that may form the basis for the clinical use of BNCT for melanoma treatment and the pharmacokinetics of BPA. In addition, the clinical results of melanoma BNCT using BPA for melanoma are discussed.

## 2. Determination of Relative Biological Effectiveness or Compound Biological Effectiveness for Melanoma Control In Vitro and In Vivo

In BNCT, thermal neutron irradiation results in a mixture of radiation types with different LETs such as high-LET radiation released from the reactions of ^10^B (n, α)^7^Li and ^14^N (n, p)^14^C; low-LET gamma rays released from the reaction of ^1^H (n, γ)^2^H; and incident gamma rays from the reactor core and constituent materials. Consequently, it is essential to clarify the relative biological effectiveness (RBE) of mixed radiation and high LET ^10^B (n, α)^7^Li and ^14^N (n, p)^14^C reactions, separately. In this article, a concept of compound biological effectiveness (CBE) is introduced. The biological effects of BNCT using two different ^10^B compounds such as BPA or BSH (sodium borocaptate) largely differ when the micro-distribution of the ^10^B-compounds is different, even if the average concentrations of the ^10^B compounds are the same. CBE is a RBE value in which differences in the micro-distribution of ^10^B are considered.

### 2.1. Effect of BNCT on Cultured Melanoma Cells Grown in Monolayer

The RBE values of thermal neutron beam and high LET reaction in cultured cells have been obtained. The B-16 murine melanoma cells were irradiated with or without ^10^B-boric acid in the culture medium. When cultured cells are grown in a monolayer in a flask, the thickness of the monolayer cells is approximately 2–4 μm, which is smaller than the range of high LET particles (7–10 μm) released from ^10^B (n, α)^7^Li and ^14^N (n, p)^14^C. Therefore, it is necessary to estimate the absorbed dose fraction, which is the energy absorbed to the cells divided by the total released energy. Fukuda et al. [15] considered monolayer cells as a finite plate and calculated the absorbed dose fraction due to the high LET reactions. The absorbed dose to the cells was calculated using Equation (1) [15,26]:

(1) $$\frac{Ep}{Q}\;=\frac{1}{2}⟦Z-\frac{d}{\left|d\right|}{\left(\frac{d}{R_{c}}\right)}^{2}+2\left(\frac{d}{R_{c}}\right)\ln\left|\frac{d}{R_{c}}\right|⟧$$

where *Q* is the total energy released by a charged particle; *Ep* is the absorbed energy at point *P*; *d* is the distance from the interface of a boron-containing region and a boron-free region; and Rc is the range of the particle. The total absorbed dose fraction to the cells was obtained by integrating the *Ep*/*Q* over the region containing the cells. Using these results, the absorbed dose to B-16 melanoma cells cultured in monolayer and irradiated with thermal neutrons with or without 5 μg/mL ^10^B boric acid was calculated. From the two survival curves with and without ^10^B boric acid, the RBE (CBE) of the ^10^B (n, α)^7^Li reaction expressed by the D_0_ ratio was 3.3 [15]. Rossini et al. [27] evaluated the RBE or CBE of M8 and Me-j cells in culture. M8 is an amelanotic cell line derived from a human cutaneous melanoma and Me-j is a pigmented cell line established from a human metastatic melanoma foci. The cells were incubated with 10 μg BPA/mL in culture medium for 2 h, and then irradiated with the reactor beam. The RBE and CBE were calculated by comparing the results with those obtained by gamma-ray irradiation. The RBE of the BNCT irradiation was 1.3 for M8 cells and 1.5 for Me-jells. The CBE of the ^10^B(n, α)^7^Li reactions was 2.1 for M8 cells and 3.0 for Me-j cells.

### 2.2. RBE of BNCT for Melanoma Control In Vivo

Hiratsuka et al. [28] investigated the RBE of the ^10^B(n, α)^7^Li reaction for the control of hamster melanoma. Hamsters bearing Green’s melanoma were intravenously administered with 10, 20, and 40 mg of BPA 8 h before thermal neutron irradiation. After the irradiation, the tumor volumes were successively measured. Growth delay time [29] was measured and used as an indicator of the radiation effect. The results were compared with those obtained with 9 MeV electrons and the CBE value of the ^10^B (n, α)^7^Li reactions for the tumor growth delay time was determined to be 2.5 (Table 1).

Coderre et al. [30] evaluated the effect of BNCT on Harding–Passey melanoma in vivo using growth delay time as an index, and found that the RBE value of BNCT irradiation was about 2.0 compared to the effect of 100 keV X-rays. The CBE value for the ^10^B (n, α)^7^Li reaction was not reported. Coderre et al. [31] also reported the in vivo effect of BNCT on B-16 melanoma cells using a morbidity index obtained from survival curves. The RBE values of the ^10^B(n, α)^7^Li and ^14^N(n, p)^14^C reactions (2.3 and 2.0, respectively) were obtained from the literature and used for the calculation of the dose in Gy-equivalent (Gy-Eq). The RBE or CBE values for melanoma control are summarized in Table 1. The value of RBE or CBE largely depends on indicators of interest, even if the same biological system is used. To specify indicators to be used, the term “endpoint” is used in the radiobiology field.

## 3. Radiobiological Basis for Human Melanoma Control by Single Irradiation

In any type of radiotherapy, the tumor must be controlled within the tolerable limits of the normal tissues. Therefore, at least two issues must be addressed in radiation dose planning for melanoma BNCT. First, what is the maximum tolerable dose to the skin by a single irradiation? This is discussed in the next section. Second, what is the curable dose of a single irradiation for malignant melanoma? The author’s group has solved these issues with the help of radiobiological data and knowledge from the study by Overgaard et al. [34].

**Figure 1:**
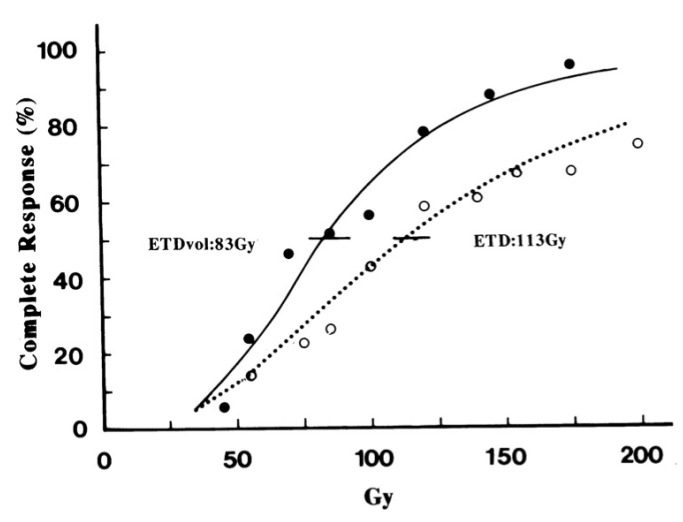
Dose response relationship showing the probability of complete response as a function of ETD or ETD_vol_. Bars indicate the 95% confidence limits of a 50% complete response probability (adopted from Overgaard J, Int J Radiat Oncol Biol Phys 1986; 5:183–192, by permission of the publisher).

No data are available on the complete curative dose of a single irradiation for human malignant melanoma. Overgaard et al. [34] analyzed the 50% tumor control dose (TCD_50_) reported in several studies on melanomas treated with X-ray at different fraction sizes and different doses per fraction. On the basis of the results, they created an iso-effect plot for a complete response (CR) of 50% as a function of dose per fraction and reciprocal total dose for melanoma cure probability. This plot yielded an α/β ratio of 2.5 Gy and extrapolated total dose (ETD) for 50% cure of melanoma. The ETD value for any treatment with different fractionation can be calculated using Equation (2):

ETD_vol_ (Gy) = {D × (d + 2.5)/2.5} × M^−0.33^ (2)

where D and d are the total dose and dose per fraction in Gy, respectively; 2.5 is the α/β ratio for melanoma; and M is the mean tumor diameter in cm and M^−0.33^ is the tumor size correction factor. Figure 1 shows the dose relationship for the probability of complete tumor response as a function of ETD with or without tumor volume correction. From this curve, an ETD_vol_ of 83 Gy and 180 Gy yielded a complete response probability of 50% and 90%, respectively. It was assumed that an ETD_vol_ of 180 Gy is the required dose for the complete cure of melanoma. Thereafter, the complete cure dose of a single irradiation can be calculated using Equation (2) under the following condition: ETD_vol_ = 180 Gy, D = d, and various tumor sizes (M). Considering that melanoma is radioresistant, it can be assumed that the tumor size is always 3 cm in diameter, even when the size is smaller than 3 cm. Then, the calculated result was 24.2 Gy-Eq for the complete cure of melanoma with a diameter of 3 cm. For dose planning, it was assumed that 25 Gy-Eq or more is required for melanoma control with a single irradiation [19,24].

## 4. RBE of BNCT for Normal Tissue (Skin) Damage

When applying BNCT for malignant melanoma, it is essential to know the maximum tolerable dose to the skin of a single irradiation because normal skin is a target of normal tissue damage. The RBE and CBE for skin damage have been analyzed in animals and humans.

### 4.1. RBE of BNCT for Animal Skin Damage

Hiratsuka et al. [35] used hamsters to analyze the effects of BNCT irradiation on skin damage. Hamsters were intravenously administered with 80 mg/kg of BPA and then irradiated with thermal neutrons 8 h later. The extent of the early skin reactions and their time course were assessed and compared with those produced by 9 MeV electron irradiation. The radiation doses of BNCT and electrons that yielded maximum skin reactions (no more than moist desquamation) by BNCT and electron were 11.1 and 24.0 Gy, respectively. The CBE of the ^10^B(n, α)^7^Li reactions for early skin damage was calculated to be 2.4. Morris et al. [36] estimated the CBE of BNCT for the skin reaction in Fisher rats after oral administration of 1500 mg/kg BPA. The 50% effective dose (ED_50_) analysis showed that the CBE for yielding moist desquamation was 3.74 ± 0.70.

### 4.2. Tolerable Dose to the Human Skin of a Single Irradiation (Estimated from the Literature)

In conventional radiotherapy, the fractionated irradiation method is used to protect against damage to normal tissues. However, BNCT is usually performed using a single dose of neutrons. As the skin is the affected normal tissue in melanoma BNCT, it is important to determine the maximum tolerable dose of a single irradiation to normal skin. Although few data are available on single-dose radiotherapy using X-rays, the data from the study by Douglas [37] were adopted. Douglas estimated the tolerable X-ray dose to the skin as a function of the fraction size and radiation filed size. Although the minimum fraction size available was 4 fractions, Douglas estimated the tolerable dose of a single irradiation by interpolation and reported that the tolerable doses of X-ray were 17.6 Gy for a 100-cm^2^ circular field, 18.6 Gy for a 75-cm^2^ circular field, and 17.3 Gy for a 6 × 8-cm rectangular field. Therefore, 18 Gy-Eq was adopted as the maximum tolerable dose to the skin of a single irradiation.

### 4.3. RBE of BNCT for Human Skin Damage

The degree of skin damage in melanoma patients who received BNCT was evaluated. Patients with melanoma were administered with 170–210 mg/kg BPA intravenously over 3–5 h, followed by BNCT. After the treatment, dermatologists carefully observed the skin appearance and damage and precisely recorded them thorough photographs. Radiation oncologists evaluated the records and determined the grade of early skin damage (skin score). In the scoring system, a score of 3 (moist desquamation) was the maximum acceptable level. Of the 22 patients, 16 patients had tolerable skin damage (score 2: seven patients, score 3: nine patients) and six patients exceeded the tolerable limits (score 4: three patients, score 5: three patients). In the literature, it is known that 18 Gy-equivalent (Eq) is the maximum tolerable dose to the skin of a single irradiation, as described above [37]. The following inequality equations can be described as

D_score 4,5_ > 18 Gy-Eq, D_score 1,2,3_ ≤ 18 Gy-Eq (3)

where D is the dose to the skin in Gy-Eq. By solving 18 inequality equations obtained from 18 BNCT cases, the RBE of the ^10^B (n, α)^7^Li reactions for human skin damage was determined to be approximately 2.5 [19,24]. In the present calculation, the RBEs of the ^14^N (n, p)^14^C and ^10^B (n, α)^7^Li reactions were assumed to be equal. The RBE value increased as the LET value increased, reached a peak at an LET value of approximately 100 keV/μm [38,39], and then decreased in an almost symmetrical manner with an upward curve. The LET of ^14^N (n, p)^14^C and ^10^B (n, α)^7^Li reactions were 60 and 230 keV/μm, respectively. Therefore, it was assumed that the RBE values of these reactions were nearly equal. The estimated RBE value of 2.5 is the only available value for early skin damage in humans, and the other reported RBE and CBE values were obtained from animal studies. Almost all groups performing BNCT worldwide use the CBE value obtained from humans [19]. The RBE and CBE values for early skin damage by BNCT are summarized in Table 2.

## 5. Pharmacokinetics of BPA in Humans

For radiation dose planning in BNCT, it is essential to know the ^10^B pharmacokinetics of BPA in the normal tissue (skin) and tumors. Fukuda [24,40] analyzed the ^10^B concentration kinetics in blood, skin, and tumor in 24 patients with melanoma who received BPA and subsequently underwent BNCT or surgery. The blood ^10^B concentration in eight patients who received 180 ± 14.9 mg/kg of BPA intravenously over 3 to 5 h and then underwent BNCT peaked at the end of the BPA infusion and then declined. The peak values varied widely, ranging from 6 to 16 μg^10^B/g blood. When the values were normalized to the peak value in each patient (^10^B concentration ratio), the variability decreased and the curves could be fitted to biphasic curves. The half-lives of the first and second components were 2.8 h and 9.2 h, respectively. The ^10^B concentration kinetics in the blood from the literature including the data of the author’s group, are summarized in Table 3. The kinetics varied widely according to the differences in the administered dose of BPA and duration of the infusion time [41].

The ^10^B concentration in tumors using resected tumors from nine patients with melanoma who received 85 or 170 mg/kg BPA intravenously was analyzed. Two patients received five consecutive subcutaneous injections of 50 mg/kg BPA, and then underwent surgery. The absolute ^10^B values in tumors varied from 5 to 22 μg^10^B/g; however, tumor-to-blood ratio (T/B) was relatively constant from 1 to 5.5 h after BPA injection, with a mean T/B ratio of 3.40 ± 0.83. Lieberman et al. [41] reported that the mean T/B ratio was 2.1 ± 0.4 at 0.75 to 4.5 h after the completion of 100 or 300 mg/kg infusion in three melanoma patients. Zhang et al. [43] reported that the T/B ratio of 2.54 ± 1.61 at 30 min or 90 min after completion of BPA infusion in two patients after the administration of 350 mg/kg BPA. The ^10^B concentrations in the skin of 15 patients with melanoma who received, with or without BNCT, was analyzed. Although the absolute values varied among the patients, the skin-to-blood ratios remained relatively constant at 1.31 ± 0.22 during the 6 h after the end of BPA administration. Zhang et al. [46] reported a skin-to-blood ratio of 1.33 ± 0.36 in two patients after the administration of 350 mg/kg BPA. Table 4 summarizes the T/B and skin-to-blood (S/B) ratios in patients with melanoma.

These reported T/B ratios suggest that the absolute ^10^B concentration in the tumor may be continuously decreased as the blood concentration decreases, when neutron irradiation is conducted after the completion of BPA infusion. This dynamic suggests that neutron irradiation should be initiated as soon as BPA administration is completed. To maintain a high tumor ^10^B concentration during neutron irradiation, Ono et al. proposed a method of continuous BPA infusion during neutron irradiation, a protocol called the two-stage infusion method [47]. With this method, the ^10^B concentration can be maintained relatively constant during neutron irradiation [41,47].

## 6. Clinical Results of BNCT for Malignant Cutaneous Melanoma

Few studies have reported the clinical results of BNCT for malignant melanoma. Mishima is a pioneer of BNCT for malignant melanoma and his group including Fukuda and Hiratsuka have played an important role.

### 6.1. Japanese Group

**Figure 2:**
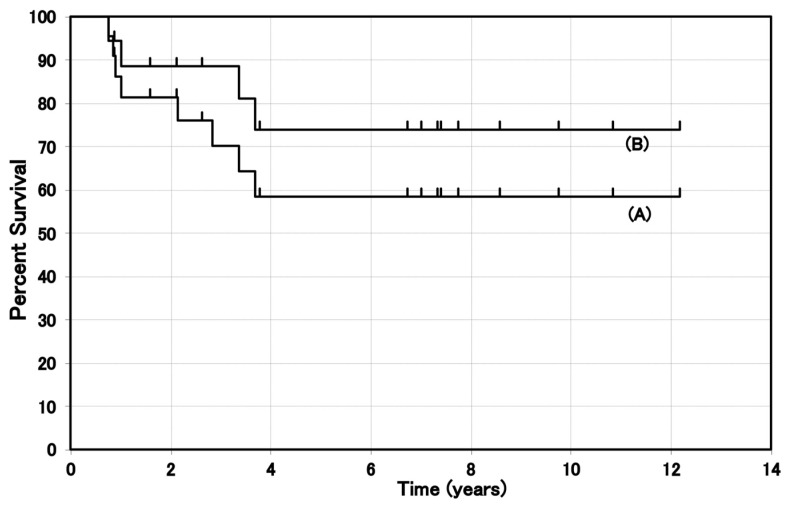
Cause-specific survival of the melanoma patients treated by BNCT. (**A**) Overall cases, (**B**) primary melanoma cases.

The first successful clinical application of BNCT with BPA for cutaneous melanoma was conducted by Mishima et al. in 1987. Then, Mishima and his group, including the author of this article, treated 22 patients with melanoma between 1987 and 2002 [24]. The dose of BPA used for BNCT ranged from170 to 210 mg/kg. The rates of complete response (CR) and partial response (PR) were 68.2% (15/22) and 23.0% (5/22), respectively. The CR rate for non-nodular melanoma was good (81.2%, 13/16), whereas the CR rate for nodular melanoma was poor (33.3%, 2/6). The response rates (CR + PR) for non-nodular melanoma and nodular melanoma were 100% (16/16) and 67% (4/6), respectively. Skin damage was an acceptable level in 19 patients except three, who developed skin ulcer or necrosis [24]. Although long-term survival was not described in this report, the original survival data of the patient were reanalyzed by the author. Of the 22 patients, 11 were alive with post BNCT survival time of 0.9 to 13, eight years at the time of evaluation (1 May 2003). Four patients with metastatic melanoma died of systemic metastases within three years after BNCT. The 5-year cause-specific survival rates were 58% and 74% for the overall and primary melanoma patients, respectively (Figure 2).

Hiratsuka et al. [25] performed BNCT on eight patients with cutaneous melanoma between 2003 and 2014. Six patients showed CR (75%) and two patients remained PR. Three patients died 5.5 to 12.6 years after BNCT. Five patients were alive with no evidence of the disease for 5.6 to 8.2 years after BNCT at the time of evaluation (20 January 2020). Although these patients were at an early stage (T_1-2_N_0_M_0_), the results were quite promising for further clinical studies. Morita et al. including Hiratsuka and Fukuda [48] performed BNCT on two patients with melanoma of the skin and two with melanoma of mucosa using 500 mg/kg BPA. Three patients had CR and one had PR. Long-term outcome was not described in this report.

### 6.2. Argentine Group

The Argentine group treated seven melanoma patients with multiple metastases in the extremities between 2003 and 2007 [49]. Since all patients had multiple metastases, BNCT was not expected to prolong survival, so the local response of each lesion in a patient was evaluated. The clinical response rate (CR + PR) on a lesion basis, not on a patient basis, was 69.3%. Patient survival ranged from four to 23 months after BNCT.

### 6.3. Other Groups

The MIT group performed BNCT in four patients with melanoma of the extremities as a phase I clinical trial between 1994 and 1996 [50]. The results showed that one patient had a complete response and two had partial responses. In one patient, the residual tumor was removed for biopsy, so tumor response could not be assessed. Yong et al. [51] reported a case of melanoma treated by BNCT using 350 mg/kg BPA. Local response of the tumor was CR, however, longer outcome and additional cases have not been reported until now. Table 5 summarizes the clinical results of BNCT on melanomas.

## 7. Discussion

BNCT for glioblastoma using BSH has been a major stream and many reviews on it have been published. While clinical BNCT for malignant melanoma using BPA was first initiated by Mishima and author’s group in 1987, it has become another stream of BNCT. However, there have been no comprehensive reviews on melanoma BNCT. Therefore, this paper has tried to describe many aspects of melanoma BNCT such as radiobiological studies in vitro and in vivo including absorbed dose estimation, pharmacokinetics, and clinical results. These matters are important for comprehensive understanding of melanoma BNCT. The points to be discussed are as follows.

### 7.1. RBE Values of BNCT Radiation and CBE of ^10^B (n, α)^7^Li Reaction Using BPA

There were only five reports on RBE or CBE in melanoma control (Table 1). The data obtained from in vivo studies are more useful than those from in vitro studies for clinical application of BNCT. Although there were only three in vivo studies on this issue (Table 1), the RBE values of BNCT mixed beam for melanoma control were relatively consistent ranging from 2.0 to 2.22. The only in vivo data for the CBE value of the ^10^B (n, α)^7^Li reaction for melanoma control was 2.5, as reported by Hiratsuka [28].

Table 1 also shows the RBE and CBE values for tumors other than melanoma. Only two data were available. Coderre [32] evaluated the effect of BNCT on Fisher 344 rats with 9L gliosarcoma in the brain. The rats were given 1500 mg/g of BPA intragastrically twice at 3-h intervals. After the rats were irradiated with thermal neutrons, brain tumors were removed and minced, and trypsinized to prepare single-cell suspension. These cells were plated in a culture flask and a colony formation assay was performed. Compared with the results from 250 keV X-rays, the CBE value of ^10^B(n, α)^7^Li reaction was deduced to be 3.8 at a survival fraction of 0.01. Suzuki et al. [33] evaluated the effect of BNCT on the C3H/He mouse bearing a SCC VII tumor implanted in the liver. After intraperitoneal administration of 75 mg/kg BPA, the mouse tumor was irradiated with thermal neutrons in vivo and then assayed in vitro using the same method described as above. The CBE value of the ^10^B(n, α)^7^Li reaction was 4.22 for the D_0_ ratio of the survival curve and the CBE value of ^10^B (n, α)^7^Li in most BNCT groups around the world, except for the author’s group, used 3.8 as reported by Coderre [32] for all kinds of tumor control. The author’s group used 2.5, which was reported in vivo by Hiratsuka [28] using hamster melanoma.

Only three studies were available for RBE or CBE of BNCT with BPA on skin damage, and only one for human skin damage [19] (Table 2). CBE values in hamster and human skin damage were similar (2.4 and 2.5), but higher in Fisher rat (3.7). The mucus membranes are more radiosensitive than skin, and the CBE value on mucosal damage has been reported to be 4.9 [52], much higher than the CBE value for skin damage.

Here, we limited our discussion to the RBE and CBE of BNCT with BPA, because BSH is not used for the BNCT of melanoma. Coderre at al. [53] reported an excellent review on RBE and CBE of BNCT with BSH and BPA. In their report, RBE and CBE with BSH and BPA for normal tissue damage such as the skin, brain, and spinal cord were summarized.

### 7.2. Optimal Dose Estimation by Single Irradiation for Melanoma Control

Since there were no data on the complete curative dose by single irradiation for human malignant melanoma, the dose was calculated with reference to the literature by Overgaard et al. [34]. Overgaard performed TC_50_ analysis using the clinical results of malignant melanoma treated by radiotherapy in a different number of fractions and different dose per fraction. This analysis yielded the α/β ratio of melanoma and the extrapolated total dose (ETD) for 50% cure of melanoma. To the best of the author’s knowledge, there are no reports of such an analysis for other types of cancers. Calculating using Equation (3), it can be inferred that 24.2 Gy would be a single dose for melanoma control with 3 cm in diameter. If the tumor size is 1 cm in diameter, the required curative dose was calculated to be 20 Gy. We adopted 25 Gy as a dose for a complete cure of melanoma. This is the first time such an estimate has been made in BNCT for any type of cancer.

### 7.3. Pharmacokinetics of BPA in Human Patients

Knowledge of the pharmacokinetics of BPA is indispensable for radiation dose planning and the determination of the timing of neuron irradiation of BNCT. Although animal experiments are important to determine detailed pharmacokinetic parameters, human studies are more useful for clinical trials of BNCT. The author summarized the pharmacokinetics of BPA in the blood, skin, and tumors in human patients [40]. Administered dose of BPA and duration of infusion time of BPA differed greatly among the BNCT groups (Table 1). Regarding the kinetics of blood concentration, BNL group analyzed the dose-dependent kinetics of BPA in detail. They administered 130, 170, 210, and 250 mg/kg of BPA and analyzed ^10^B concentration kinetics in the blood. Maximum value of ^10^B in the blood at the end of infusion ranged from 13.1 to 22.1 μg/g blood, which was approximately a linear function of the administered dose of BPA [42].

As described above, when neutron irradiation was conducted after completion of a single infusion of BPA, the absolute ^10^B concentration in the tumor may be continuously decreased as the blood concentration decreases. To overcome this drawback, Ono proposed a two-stage infusion method of BPA [47]. In this method, neutron irradiation was performed in the second stage. At the end of first stage after 400 mg/kg administration over 2 h, a mean ^10^B value in the blood was 26.8 ± 5.5 μg^10^B/g blood. At the end of second-stage infusion of 100 mg/kg over one hour, a mean ^10^B value in the blood was 26.4 ± 5.9 μg/g blood. Blood ^10^B concentration just before and just after neutron irradiation were almost the same. At the present time, most of the BNCT groups including Japan, Taiwan, and China have adopted this method.

All these data and knowledge of the pharmacokinetics described here were integrated and used for optimization of treatment planning for each patient.

### 7.4. Clinical Results

Since Mishima and his group (including the author) played an important role in the development of melanoma BNCT, most of the clinical reports are from Japan [3,4,18,19,20,21,22,23,24,25,40,41,48]. The only paper from Japan (Hiratsuka) described the long-term survival of the patients [25]. Patients survived from 5.5 to 12.6 years after BNCT. Fukuda reported the clinical results of BNCT in 22 melanoma patients [24]. Long-term survival rates were not given in this report, but the original survival data for these patients were reanalyzed by the author. The case specific 5-year survival rates were 58% and 74% for all cases and for the primary melanoma patients, respectively. These results suggest that BNCT of melanoma is clinically promising.

An Argentine group reported the clinical results of seven patients with melanoma having multiple metastases. The reported survival time ranged from four to 36 months, as prolonged survival in these patients is not expected. To date, no additional studies from Argentine have been reported.

### 7.5. BNCT of Cancer as Clinical Practice

As described in the introduction, the Japanese government approved the health insurance coverage of cyclotron-based BNCT for head and neck cancer in 2020. This approval is first in the world as clinical practice. Hirose et al. presented the results of an open-labeled phase II trial for 21 patients with recurrent or locally advanced head and neck cancers [54]. The rate of CR, PR, and overall response (CR + PR) rate were 24%, 48%, and 71%, respectively. The 2-year overall survival was 85% and the median local progression free survival was 11.5 months. Based on the data, the Japanese government has approved this therapy. Recently, a phase I/II clinical trial of cyclotron-based BNCT for recurrent glioblastoma was completed in Japan, and approval of the therapy is under consideration. Currently, a clinical trial of BNCT for hemangiosarcoma and melanoma is ongoing in Japan.

## 8. Conclusions

In this paper, we presented a logical approach to clinical trials of BNCT for melanoma based on radiobiological data and knowledge. The results of the clinical trials are very positive and BNCT of melanoma is promising as a clinical practice.

## Figures and Tables

**Table 1:** RBE and CBE values of melanoma control by BNCT using BPA.

Tumor Line	BNCT_beam_	^14^N (n, p)^14^C	^10^B (n, α)^7^Li	Endpoint	Reference
B-16 (vitro)	2.55	-	3.3	D_0_ ratio	Fukuda, 1987 [15]
M8 (vitro)	1.3	1.38	2.1	Ratio at SF 0.001	Rossini, 2014 [27]
Me-J (vitro)	1.5	1.75	3.0	Ratio at SF 0.001	Rossini, 2014 [27]
Green’s melanoma (vivo)	2.22	3.0	2.5	Growth delay time	Hiratsuka, 1989 [28]
Harding–Passey (vivo)	2.0	-	-	Growth delay time	Coderre, 1988 [30]
B-16 (vivo)	2.0	(2.0) ^1^	(2.3) ^1^	Morbidity index	Coderre, 1991 [31]
9L-gliosarcoma (vivo)	2.3	3.2	3.8	Ratio at SF 0.001	Coderre, 1993 [32]
SCC VII (vivo)	2.79	-	4.22	D_0_ ratio	Suzuki, 2000 [33]

^1^: Adopted from a literature. B-16: murine (C57Black) melanoma; M8: human amelanotic melanoma; Me-j: human pigmented melanoma; Green’s melanoma: hamster melanoma; Harding–Passey: murine (Balb/c) melanoma; 9L-gliosaracome: rat (Fishier) gliosarcoma; SCC VII: murine (C3H/He) squamous cell carcinoma; D_0_: slope of the survival curve; SF: survival fraction.

**Table 2:** RBE and CBE values for early skin damage by BNCT using BPA.

System	BNCT_beam_	^14^N (n, p)^14^C	^10^B (n, α)^7^Li	End Point	Reference
Hamster	2.16 ± 0.06	2.9 ± 0.04	2.4 ± 0.06	Moist desquamation	Hiratsuka, 1991 [35]
Fisher rat	-	3.50 ± 0.23	3.74 ± 0.70	Moist desquamation	Morris, 1994 [36]
Human	-	2.5	2.5	Moist desquamation	Fukuda, 1994 [19]

**Table 3:** ^10^B concentration kinetic in the blood following administration of BPA with various doses and various infusion times.

Group	BPA Dose (mg/kg)	Infusion Time (h)	Number of Patients	Maximum Value (μg/g)	T_1/2_ (1st) (h)	T_1/2_ (2nd) (h)	Reference
Japan	500 iv	2–3	9	36.9 ± 6.9	0.8	6.7	Fukuda, 2020 [41]
	180 ± 14.9 iv	3–5	8	9.4 ± 2.6	2.8	9.2	Fukuda, 1999 [40]
	174 ± 7.0 iv +30 sc	3–5	7	7.4 ± 2.1	3.3	9.0	
	85 iv	2–3	7	6.8 ± 1.2	3.7	10.0	
USA	250 iv	2	3	22.1 ± 3.4	1.2	8.2	Elowitz, 1998 [42]
	210 iv	2	3	17.3 ± 3.7	n.a	n.a	
	170 iv	2	4	14.2 ± 2.2	n.a	n.a	
	130 iv	2	5	13.1 ± 1.9	n.a	n.a	
Germany	100 iv	1	3	9.5 ± 0.8	0.7	7.3	Wittig, 2009 [43]
Argentina	300 iv	1.5	3	22.1–25.3	0.3	11.0	Lieberman, 2004 [44]
	100 iv	1–1.5	3	5.5–9.8	n.a	n.a	
Sweden	900 iv	6	18	24–50	1.4	12	Capala, 2003 [45]

T_1/2_ (1st), T_1/2_ (2nd): half-lives of first and second components of biphasic blood clearance curve, respectively; iv: intravenous infusion; sc: subcutaneous administration; n.a: not available (data adopted form Fukuda, H., Hiratsuka, J., ARI 2020, 166, 109308).

**Table 4:** Skin-to-blood (S/B) and tumor-to-blood (T/B) concentration ratios following administration of BPA in melanoma patients.

Group	BPA dose (mg/kg)	Infusion Time (h)	Skin/Blood (Number of pts)	Tumor/Blood (Number of pts)	Hours after the End of Infusion	Reference
Japan	85, 170 iv	3–5	1.31 ± 0.22 (15)	3.40 ± 0.83 (11)	1.0–5.5	Fukuda, 1999 [40]
Argentina	100, 300	1.5	-	2.1 ± 0.4 (3)	0.75–4.5	Lieberman, 2004 [44]
China	350	1.5	1.33 ± 0.36 (2)	2.54 ± 1.61 (2)	0.5, 1.5	Zhang, 2020 [46]
	90	1.5	1.05 (1)	1.48 (1)	0.75	

**Table 5:** Clinical results of BNCT for cutaneous melanoma using BPA.

Reference (Period of Clinical Study)	Stage	BPA dose (mg/kg)	Number of Patients	Tumor Response	Long-Term Survival
Fukuda, 2003 [24] (1987–2002)	II–IV	170–210 iv	22	CR 68.2%, PR 23.0%	5-year survival 58% ^#^
Busse, 2006 [46] (1994–1996)	Recurrent melanoma	400 (oral)	3	CR 25%, PR 75%	Not available
Menendez, 2009 [49] (2003–2007)	IV Multiple metastases	300 iv	7	Lesion-based CR + PR 69.3%	36 months, 3 dead
Hiratsuka, 2020 [25] (2003–2014)	T_1-2_N_0_M_0_	500 iv	8	CR 75%, PR 25%	Survived 5.5–12.6 years, 3 dead

#: calculated using unpublished data of the author; Iv: intravenous infusion; oral: oral administration.

## Data Availability

The data are not publicly available due to data sharing rule among co-authors of the melanoma BNCT project.
